# Clinical Application of Custom Neck Collar with Negative Sensory Feedback in Children with Intractable Torticollis

**DOI:** 10.3390/children11081001

**Published:** 2024-08-16

**Authors:** Jeewon Yoon, Rayu Yun, Sungchul Huh, Jisoo Baik, Jae Meen Lee, Soo-Yeon Kim

**Affiliations:** 1 Department of Rehabilitation Medicine, Pusan National University Yangsan Hospital, Yangsan 50612, Republic of Korea; chamo125@pusan.ac.kr (J.Y.); dryunry@pusan.ac.kr (R.Y.); dr.huhsc@pusan.ac.kr (S.H.); 2 Research Institute for Convergence of Biomedical Science and Technology, Pusan National University Yangsan Hospital, Yangsan 50612, Republic of Korea; zisoo@pusan.ac.kr; 3 Department of Neurosurgery, Pusan National University Hospital, Busan 49241, Republic of Korea; geosung1@pnuh.co.kr; 4 Department of Rehabilitation Medicine, Pusan National University School of Medicine and Research Institute for Convergence of Biomedical Science and Technology, Pusan National University Yangsan Hospital, Yangsan 50612, Republic of Korea

**Keywords:** torticollis, orthosis, sensory, feedback

## Abstract

Background/Objectives: The aim of this study was to investigate the effect of a custom neck collar with negative sensory feedback for the treatment of torticollis that was previously unresponsive to conservative or surgical treatment. Methods: Twenty-four children diagnosed with unresponsive or intractable torticollis were enrolled in this two-stage, single-arm study. The ipsilateral aspect of the orthosis is adjustable in height and designed to provide support between the clavicle and the mandibular angle on the tilted side. In stage 1 (the adjustment period), the orthosis with a smooth surface was applied for 2 h per day for 3 months. In stage 2, a rough surface with a hook-and-loop fastener (Velcro©) was attached to the collar, and it was worn for a further 2 h a day for 3 months. Twenty children (mean age 63.95 ± 13.44 months) were included in the analysis. Results: The mean torticollis angle was 17.60 ± 5.65° (mean ± SD) at baseline; 14.15 ± 3.62° directly after stage 1; and 6.00 ± 3.67° directly after stage 2 (X^2^ = 36.685, df = 19, *p* = 0.000). Conclusions: This study demonstrated the feasibility, therapeutic effect, and safety of a novel tactile feedback orthosis for the treatment of children with torticollis. The use of a custom neck collar with negative sensory feedback may be a viable therapeutic option for the treatment of unresolved or intractable torticollis.

## 1. Introduction

Torticollis, also called ‘wryneck’, is a common pediatric musculoskeletal condition associated with abnormal asymmetric head or neck posture [1]. With an incidence from 0.3% to 1.3%, about 70% of head and neck abnormalities that appear in infancy or childhood are congenital muscular torticollis (CMT) [2,3]. The term torticollis, which means ‘twisted neck’ in Latin, was first defined by Tubby in 1906, and since then, more than 80 different causes have been described [4,5,6]. This condition is classified based on the position of the head and the neck in relation to the body plane. Torticollis is defined by rotation on the transverse plane; laterocollis is a one-sided tilt on the coronal plane; anterocollis is a forward tilt on the sagittal plane; and retrocollis is a backward tilt on the sagittal plane [7]. Rotation and tilting are often simultaneously present.

Congenital muscular torticollis is further categorized as sternocleidomastoid (SCM) with mass, muscular torticollis [8], or postural torticollis [9]. Congenital muscular torticollis with SCM mass is the most severe form of the condition. It becomes apparent at birth and presents as fibrotic thickening of the SCM muscle with limited passive range of motion (PROM). Muscular torticollis is distinguished by SCM muscle tightness and limited PROM of the neck without SCM mass [8,10,11] and is moderately severe. Postural torticollis, the mildest presentation, is generally diagnosed around the time that children usually achieve head control and is evidenced by a postural preference without muscle mass or restricted PROM. The prognosis of CMT is generally favorable, with 70% to 90% of patients normalizing within the first year of life after physical therapy [9,12]. Although reports of refractory CMT are not common, unresolved CMT in older children or adults may require botulinum toxin injection or surgical treatment. Surgical outcomes after the age of 1, and even in adulthood, have been reported as favorable [13,14,15,16].

Torticollis can be caused by issues other than muscle problems. The incidence of ocular torticollis is 5.6% in general ophthalmological practice and 3.19% in pediatric ophthalmological practice [17,18]. In most cases, ocular torticollis can be corrected through surgical treatment, but postural abnormalities often persist even after surgery, in which case, ongoing exercise (including PROM), symmetrical movement, and environmental adaptation are necessary to prevent further deformities [19].

In infants, if the torticollis posture persists for an extended duration, plagiocephaly occurs, resulting in deformation [3,12,20,21]. Because torticollis often cascades to scoliosis in children and adolescents [22,23], postural abnormalities that persist until preschool and school age require postural correction.

The treatment of torticollis depends on the specific etiology of a patient’s condition, but physical therapy (stretching, strengthening, and developmental facilitation) and repositioning are generally the first-line options. If physical therapy or surgical treatment does not provide sufficient improvement, an orthosis can be used as an adjunctive treatment to correct head and neck posture. According to the American Physical Therapy Association Academy of Pediatric Physical Therapy Clinical Practice Guidelines, the effectiveness of a neck orthosis for torticollis showed level V evidence, which is low [9].

Most previous studies report the use of neck orthoses to provide passive support for abnormal head posture in children with torticollis. The use of the Tubular Orthosis for Torticollis (TOT) collar, a soft foam collar, and a custom fabricated neck collar has been studied in this population, but the isolated effect of a neck orthosis for the treatment of torticollis has not yet been clearly established [24,25,26,27,28,29,30].

Especially in children with torticollis, postural correction through active movement is necessary for the child to properly acquire and establish vertical orientation of their body structures. In children and adults, active movement can be self-induced, but continuous direction or sensory feedback is often necessary for corrective exercises to be effective. Studies in scoliosis have demonstrated the effectiveness of providing sensory feedback and inducing active movement [31,32]. As of yet, there have been no similar studies for the treatment of torticollis.

There are no studies investigating the use of a neck orthosis with sensory feedback to induce active neck movement. This study aimed to investigate the effectiveness of a custom soft neck collar with negative sensory feedback for the treatment of children with torticollis who have previously been unresponsive to physical therapy and/or surgical intervention.

## 2. Materials and Methods

### 2.1. Participants

This study was conducted in accordance with the principles of the Declaration of Helsinki, and all patients’ caregivers provided written informed consent prior to their child’s enrollment. The study protocol was approved by the institutional review board (IRB No. 05-2020-033, February 2020) of Pusan National University Yangsan Hospital.

This prospective study was conducted at a single tertiary children’s hospital from March 2021 to February 2024. Patients were recruited from the Department of Pediatric Rehabilitation Medicine at Pusan National University Yangsan Hospital in Yangsan, South Korea. The eligibility criteria included (a) the diagnosis of non-paroxysmal torticollis of muscular, ocular, or postural origin; (b) a head tilt angle > 5°; (c) aged 4 years or older; and (d) having exercised sufficiently for more than 6 months according to the clinical practice guideline for torticollis [9] without achieving improvement in the torticollis angle < 5° and/or with caregiver dissatisfaction. The exclusion criteria included patients with (a) intellectual disabilities; (b) neurological deficits; (c) a range of motion of cervical rotation < 60°; (d) osseous torticollis (skeletal structural abnormality of congenital, traumatic, or inflammatory origin); and (e) the ongoing application of other treatments (physical therapy, other braces, surgery) during the study period. All patients who met the inclusion criteria during the enrolment period were invited to participate.

Patients with postural torticollis (PT group) were defined as having an absence of evidence of muscular abnormalities on neck ultrasound imaging and no neck muscle tightness. Patients with congenital muscular torticollis (CMT group) were defined as having evidence of muscular lesions on neck ultrasound imaging and/or neck muscle tightness and a history of surgery targeting the neck muscles. Patients with ocular torticollis (OT group) were defined as having been diagnosed with ocular problems, such as strabismus, which had previously been surgically corrected.

### 2.2. Design of Neck Collar

**Figure 1:**
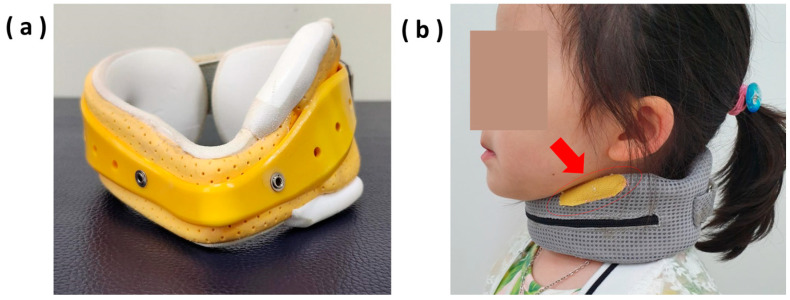
Orthosis construction: (**a**) the neck collar constructed from foam sponge which overlays a polypropylene central structure to maintain the collar’s shape without putting pressure on the larynx; (**b**) the outside of the collar finished with a mesh material. The arrow indicates the hook-and-loop fastener (Velcro^®^) tape attached to the area of contact with the mandibular angle on the tilted head side to provide negative sensory feedback.

The neck collar was designed with a donut shape and a fastener located at the back. The fastener was made with a hook-and-loop fastener (Velcro^®^, Velcro USA Inc., Manchester, NH, USA) strap so that children can unfasten and easily remove the neck collar on their own, eliminating the risk of strangulation. It was constructed from a foam sponge which overlayed a polypropylene central structure and was used to maintain the collar’s shape without putting pressure on the larynx, even when worn for a long time. The outside of the collar was finished with a removable and washable mesh material for comfort and breathability. The sides of the collar were asymmetrical in height; the height of the side where the head tilted corresponded with the distance between the clavicle and the mandibular angle, and the height on the opposite side was about 30% lower than that. The height could be adjusted by adding pads inside. By attaching hook-and-loop fastener (Velcro©) tape to the orthotic at the point where the mandibular angle tilted, a rough surface was created to provide mild tactile discomfort. The negative sensory feedback causes the patient to actively tilt their head in the opposite direction (Figure 1). This novel brace was developed and characterized by the author, Dr Kim, at Pusan National University Yangsan Hospital. It was patented in Korea in October 2019 (patent no.: 10-2040375).

### 2.3. Study Protocol

This was a single-arm, two-stage study. In stage 1, the adaptation stage, the orthosis without a rough surface attachment was applied continuously for 2 h per day, 5 days per week, over a period of 3 months. In stage 2, the rough surface was added to the orthosis to provide sensory feedback. The collar was again worn continuously for 2 h per day, 5 days per week, for a further 3 months. The time that the neck collar was worn was recorded by the caregivers in a diary daily. Participants were excluded from analysis if the brace was worn for less than 2 h a day or for less than 5 days a week for a total of 3 weeks or more.

**Figure 2:**
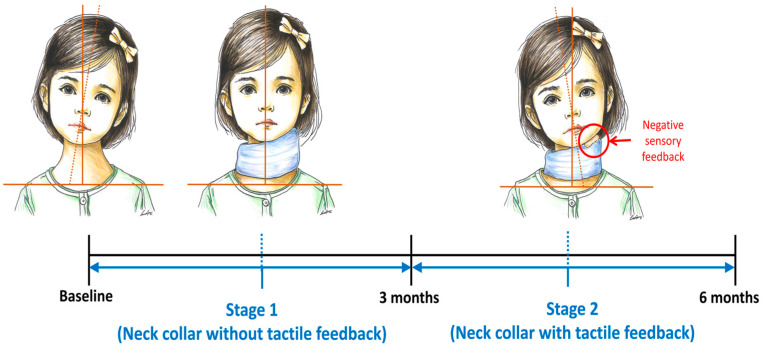
Study protocol.

There was no rest period between stage 1 and stage 2 (Figure 2). The patients and their caregivers were educated at baseline about how to perform neck exercises for active stretching of the ipsilateral neck muscles and strengthening exercises for the contralateral neck muscles. At the beginning of stage 2, they were also instructed to avoid contact with the rough surface on the neck collar.

The primary outcome was the degree of torticollis angle correction (the change in the angle of head tilt), which represents the treatment effect. The torticollis angle was evaluated at baseline, directly after stage 1, and directly after stage 2. Caregivers also reported their level of satisfaction with the treatment following stage 2 via a questionnaire. Safety was evaluated at the end of stage 1 and stage 2 by parent report and physical inspection of the lower jaw where the rough surface was attached to the orthotic.

### 2.4. Outcome Measurement

#### 2.4.1. Inclination of the Head (Torticollis Angle)

Inclination of the head was measured by two skilled physiatrists using a goniometer and still photography. We conducted an earlier study to investigate the quantitative measurement of torticollis in which the absolute agreement between the neck angles measured by the same two examiners was high (ICC value 0.997) [33].

**Figure 3:**
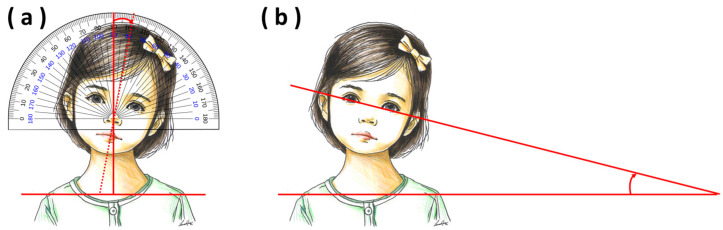
(**a**) Goniometer method. (**b**) Still photography method.

Using the goniometer method, the patient was placed in a sitting position, and the goniometer was centered on the sternum. Then, the slope of an imaginary extension line of the philtrum was measured, based on a line parallel with both clavicles (Figure 3a) [34,35]. Using the photograph approach, the patient was placed in a sitting position, and photographs were taken using a digital camera while the patient was instructed to look behind the person taking the picture. Photographs of each child were printed, and the angle formed by the line connecting the two eyes and the line connecting the acromion was measured (Figure 3b) [36]. To reduce measurement bias, the process was repeated in triplicate (with three different photographs), and the average of the three values was obtained as the final value. Finally, the average of the value from the goniometer method and the still photography method was determined and used as the representative value.

#### 2.4.2. Brace Tolerance Assessment

After completing stage 2, the patient’s tolerance and satisfaction with the orthosis were assessed with the caregivers using a modified version of the questionnaire reported by Shin et al. to assess satisfaction with spinal bracing [37]. The questions included items to assess patient posture, ease of putting on the brace, and overall success of the brace. The answer forms of the original questionnaire were transformed into a numeric rating scale, with 10 representing the best improvement or success, and 0 representing the least successful outcome [38].

#### 2.4.3. Safety Assessment

Safety was evaluated at the end of stage 2 by parent report and physical inspection. Major adverse events were defined as difficulty breathing due to pressure on the larynx when wearing the orthosis and skin lesions such as infection or bleeding at the area in contact with the orthosis.

### 2.5. Statistical Analysis

All statistical analyses were performed using Python (version 3.10.12) within the Visual Studio Code environment, leveraging libraries such as pandas, numpy, scipy, statsmodels, and matplotlib. The Shapiro–Wilk test was used to determine if the data followed a normal distribution and was applied to all continuous variables. The non-parametric Wilcoxon Signed-Rank Test was used to compare baseline angle measurements with follow-up measurements at 3 and 6 months. To compare differences between two independent groups, the Mann–Whitney U test was utilized. For comparing more than two groups, the Kruskal–Wallis test was employed. To analyze repeated measures data, the Friedman test was utilized. The Spearman’s Rank Correlation test was used to assess the strength and direction of association between two ranked variables and was applied to evaluate potential relationships between various clinical measurements. The optimal initial angle threshold for the best treatment outcomes was determined using ROC curve analysis and Youden’s Index. All significance was set at *p* < 0.05.

## 3. Results

### 3.1. Characteristics of Subjects

A total of 24 children were enrolled in this study, and 4 children withdrew due to poor cooperation or follow-up loss and did not undergo one safety or efficacy assessment. Two of the four children discontinued wearing the orthosis in stage 2 due to poor tolerability with negative sensory feedback. Therefore, 20 children (11 boys and 9 girls, mean age 63.95 ± 13.44 months) participated in this study. The demographic data are shown in Table 1. Five patients were in a postoperative state of ocular torticollis, four patients were in a postoperative state of congenital muscular torticollis, and eleven patients had postural torticollis.

### 3.2. Therapeutic Effect of Custom Neck Collar without and with Negative Sensory Feedback on Torticollis Angle

**Figure 4:**
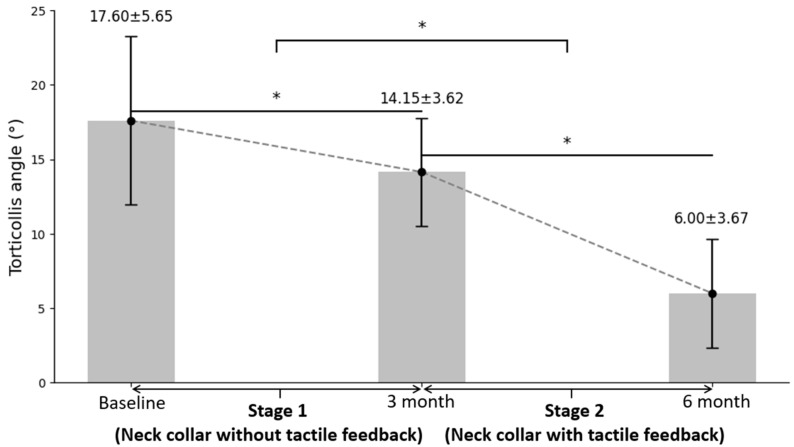
Treatment effect of custom neck collar on torticollis angle. Asterisk (*) means *p* < 0.05. Each value is presented as mean ± standard deviation.

A considerable improvement in the torticollis angle was revealed after stage 2 (Figure 4). At baseline, the mean angle was 17.60°; after stage 1, the mean angle improved to 14.15°; and after stage 2, the mean angle improved to 6.00° (X^2^ = 36.685, df = 19, *p* = 0.000), which was significantly higher than the angle at both baseline and the end of stage 1. When comparing changes in the torticollis angle with and without negative sensory feedback, the period during which the neck collar with tactile feedback was used (stage 2) resulted in a more drastic improvement compared to the period during which the neck collar without tactile feedback was used (stage 1, Z = 3.990, *p* = 0.000).

Table 2 shows the changes in the torticollis angle by diagnosis (postoperative state of congenital muscular torticollis, postoperative state of ocular torticollis, and postural torticollis). Significant improvements in the torticollis angle were observed in all groups, but the postural torticollis group showed the largest improvement from baseline (χ^2^ = 21.140, *p* = 0.000).

### 3.3. Impact of Characteristics on Treatment Effect

**Figure 5:**
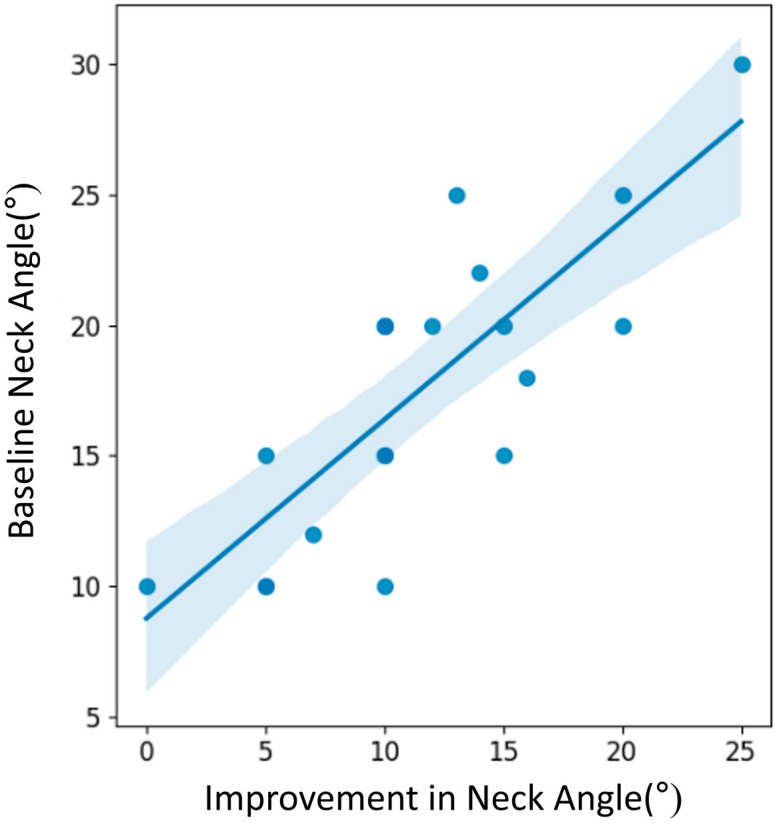
The relationship between the baseline neck angle and the improvement in neck angle. The regression line indicates the trend, with the shaded area representing the confidence interval. As the regression line appears to have an upward trend, it indicates that a larger baseline neck angle tends to be associated with a greater improvement in neck angle.

Torticollis angle correction was significantly better in patients with a higher baseline neck angle (r = 0.751, *p* = 0.000) (Table 3). Age when starting treatment, gender, and side of tilt did not affect the treatment effect. The scatter plot in Figure 5 shows a positive correlation between the baseline neck angle and the improvement in neck angle.

**Figure 6:**
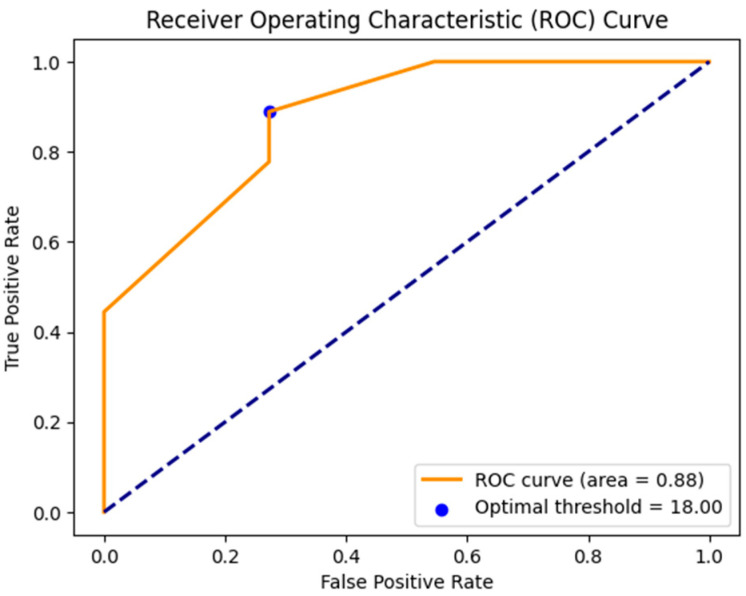
The ROC curve for determining the optimal baseline angle threshold in torticollis treatment. The blue dot indicates the point where the sum of sensitivity and specificity is maximized.

Figure 6 illustrates the ROC curve used to determine the optimal initial angle threshold for predicting effective treatment outcomes in patients with torticollis. The area under the ROC curve was 0.88. Additionally, the optimal threshold was 18 degrees.

### 3.4. Treatment Satisfaction

**Figure 7:**
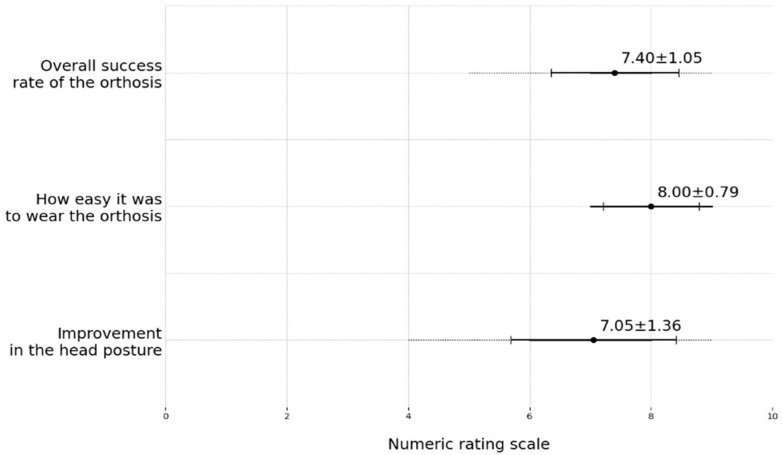
Caregiver questionnaire results. Zero presents the least favorable outcome and ten indicates the best improvement or satisfaction.

After stage 2, the majority of patients’ caregivers reported that they were satisfied with their child’s corrected head posture (Figure 7). The mean reported ‘overall success rate of the orthosis’ was 7.40 ± 1.05, the mean reported ‘ease of wearing the orthosis’ was 8.00 ± 0.79, and the mean reported ‘improvement in head posture’ was 7.05 ± 1.36.

### 3.5. Adverse Events

Minor complications occurred in only three patients who were reported to have experienced minor skin lesions with transient redness on the mandibular angle at the site of contact with the rough surface of the orthotic. There were no reports of major complications.

## 4. Discussion

In the present study, we successfully used a neck collar to induce active movement in patients whose torticollis was not resolved through conservative physical therapy or surgical treatment. The custom neck collar with negative sensory feedback provides minimally noxious stimuli to induce active movement away from the tilted head position.

The results of our study demonstrate that it is effective, safe, and well tolerated, and has a high caregiver satisfaction rating. The caregivers’ mean numerical satisfaction rating was high, and the patients’ adaptability was good, with a low dropout rate of 16% (4 of 24) and the absence of any major adverse events or safety issues. Children with postural torticollis demonstrated a greater treatment effect compared with children in a postoperative state of ocular torticollis and those in a postoperative state of congenital muscular torticollis. We believe that in patients with postural torticollis, since there was no obvious structural problem with the muscles, the correction effect was greater than in the group of children with structural problems, even if the causative disease had been corrected. Patients with more severe torticollis at baseline showed the greatest correction effect. The optimal initial neck angle threshold for predicting effective treatment outcomes was 18° from the vertical line. Because meaningful changes in angle can be expected in patients with more severe conditions compared to mild conditions, the application of this intervention may be a valuable treatment option for children with large inclination angles.

In cases where non-face-to-face treatment is required, such as during the COVID-19 pandemic or when patients have difficulty frequently visiting the hospital, the application of a tactile feedback orthosis may be an appropriate treatment option as an adjunct to a home-based exercise regimen [39]. This particular custom neck collar with negative sensory feedback is a simple but novel brace developed to provide sensory stimulation to prompt the patient to actively hold their head in a corrected posture. Future advancements in the design of the neck collar may include installing a sensor to detect pressure and head tilt and provide subsequent biofeedback through vibration, etc. In this study, objective monitoring was limited as it relied on the caregivers’ recording compliance with wearing time (5 days a week, 2 h a day). In future research, attaching a sensor to the brace and collecting data through an application will enable the objective collection of wear time to monitor compliance.

This study, conducted to assess the feasibility and safety of the orthotic, included children aged 4 years or older because it was expected that children in this age group could induce active movement on their own by following age-appropriate instructions. In other studies, braces were applied at an earlier age, starting from 4 months [24,26,40]. Future research is needed on a wider age range, including younger age groups, to validate the results of the current study in younger patients.

Studies investigating the use of the TOT neck collar and other collars required the patient to wear the orthosis for 3 to 6 h a day [24,26]. In the current study, considering the compliance of young children with noxious stimuli, the wearing time was set to 2 continuous hours a day, for 5 days a week. However, additional research is needed to optimize this protocol (wearing time and frequency).

This study has several limitations. First, it was conducted with a small sample size in a single group of patients who continuously wore the device for a period of 6 months, making it difficult to determine the isolated effect of negative sensory feedback. Through this study, the feasibility and safety of the custom neck collar with negative sensory feedback were confirmed. Whether the effect is due to passive support or active movement in response to negative feedback needs to be proven through larger, randomized, controlled studies. Second, there were no long-term follow-up data collected. A long-term follow-up study is needed to determine whether the treatment effect is maintained. Third, in this study, head tilt angle was measured two-dimensionally using a goniometer and still photography, which are measurement methods commonly used in clinical practice. However, torticollis is a three-dimensional (3D) condition and, in general, shows head rotation and tilting in combination. In a previous study, we proposed implementing a 3D angle measurement system to determine the extent of torticollis and evaluate the effectiveness of an intervention in the clinical context [33]. We propose that further 3D angle measurement approaches may be employed in the future to more accurately determine the effectiveness of the intervention.

The present study was conducted on young children with postural torticollis, treated muscular torticollis, and treated ocular torticollis, and excluded those with structural problems. Future research is needed to determine whether the application of the custom neck collar with negative sensory feedback is effective in groups with a more diverse etiology and in patients with structural problems.

## 5. Conclusions

This study is the first to apply a sensory biofeedback neck collar to induce active movement for the treatment of young children with torticollis. We demonstrated the therapeutic effect, safety, and feasibility of the custom neck collar. Although larger studies with a controlled design covering a wider range of ages and etiologies are needed, this brace is valuable as a simple and cost-effective treatment strategy that can be easily applied in clinical practice. A custom neck collar with negative sensory feedback may be a treatment option for children with unresolved or intractable torticollis, especially those with a more severe baseline head tilt angle.

## Figures and Tables

**Table 1:** Demographic data of subjects (N = 20).

Variables		Mean ± SD
Age (months)		63.95 ± 13.44
Baseline torticollis angle		17.6 ± 5.65
		N (%)
Gender	Male	11 (55.0)
	Female	9 (45.0)
Side of lesion	Right	12 (60.0)
	Left	8 (40.0)
Diagnosis	Postural torticollis	11 (55.0)
	Postoperative state of ocular torticollis	5 (25.0)
	Postoperative state of congenital muscular torticollis	4 (20.0)

**Table 2:** Torticollis angle changes by diagnosis.

Diagnosis	Baseline Angle (Mean ± SD)	Angle at End of Stage 1 (Mean ± SD)	Angle at End of Stage 2 (Mean ± SD)	SS	df	MS	Χ^2^	*p*
PSCMT	18.75 ± 4.79	14.75 ± 3.77	6.75 ± 5.38	27	2	13.5	8.000	0.018 *
PSOT	15.60 ± 5.18	12.80 ± 2.77	6.40 ± 3.51	75	2	37.5	7.600	0.022 *
PT	18.09 ± 6.35	14.55 ± 4.03	5.55 ± 3.39	1056	2	528	21.140	0.000 *

Asterisk (*) means *p* < 0.05. PSCMT (postoperative state of congenital muscular torticollis), PSOT (postoperative state of ocular torticollis), PT (postural torticollis), SD (standard deviation), SS (Sum of Squares), df (Degree of Freedom), MS (Mean Square), χ^2^ (Chi-Square), and *p* (*p*-value).

**Table 3:** Impact of properties on treatment outcome.

Variables	Improvement in Neck Angle	
	r-Value	*p*-Value
Age when starting treatment (months)	0.026	0.915
Baseline neck angle (°)	0.751	0.000 *
Gender	0.292	0.211
Side of tilt	2.216	0.361
Diagnosis	−0.081	0.736

Asterisk (*) means *p* < 0.05.

## Data Availability

The data presented in this study are available on request from the corresponding author. The data are not publicly available due to privacy or ethical restrictions.
